# Topiramate-Induced Persistent Eyelid Myokymia

**DOI:** 10.1155/2016/7901085

**Published:** 2016-05-17

**Authors:** Mohammadrasoul Khalkhali

**Affiliations:** Department of Psychiatry, Shafa Hospital, Guilan University of Medical Sciences, Rasht 4165863795, Iran

## Abstract

*Background*. Topiramate (TPM) is a psychotropic drug, which is used mainly as an antiepileptic drug and now over the years is used for a wider range of indications, including migraine prophylaxis and binge eating disorders. Although ocular side effects of Topiramate have been frequently reported, neuroophthalmologic manifestations such as myokymia are rarely reported.* Case Presentation*. This case report presents a case of a 47-year-old woman who had begun TPM for binge eating problem. She developed unilateral long standing lower eyelid twitching, which progressed to upper eyelid and eyebrow at the same side. The patient was not a smoker or excessive alcohol or caffeine abuser. Increasing the resting time and changing life style made no significant changes in her eyelid twitching. There was no definite evidence by neuroimaging and clinical or laboratory evaluations causing eyelid myokymia. The symptoms resolved with discontinuation of TPM.* Conclusion*. Although eyelid myokymia is a benign and self-limited condition, it sometimes becomes a source of distress in chronic long standing cases. Physicians should be aware of the neuroophthalmologic side effects of this drug.

## 1. Introduction

TPM is used mainly as an antiepileptic drug, both as monotherapy and as an adjunct in the control of partial and generalized epilepsy in children and adults [1]. Its effectiveness in trigeminal and postherpetic neuralgia, migraine prophylaxis, bipolar disorders, alcohol and tobacco use disorder, depression and obesity, binge eating disorders, essential tremor, obsessive-compulsive disorder, idiopathic intracranial hypertension, neuropathic pain, and Tourette's disorder has also been reported [2, 3]. The combination of phentermine/TPM was approved by FDA in 2012, with a Risk Evaluation and Mitigation Strategy (REMS) [3]. TPM induces weight loss not only by decreasing food intake but also by increasing energy expenditure, possibly through decreased efficiency of nutrient utilization in animal models, but TPM appetite regulation and weight loss induction mechanisms are not well known in human [4]. The most common adverse events related to TPM are sedation, drowsiness, dizziness, cognitive disturbance, unusual tiredness or weakness, menstrual problems, and vision problems. Paresthesia, nausea, abdominal pain and weight loss, headache, and depression are less common [2, 5, 6]. Ocular side effects of TPM are hyperemia, mydriasis, acute angle closure glaucoma, ocular pain, headache, uveitis, visual field defects, acute onset myopia, retinal hemorrhage, suprachoroidal effusions, and scleritis. Neuroophthalmologic manifestations such as blepharospasm, myokymia, and oculogyric crisis are rarely reported [2, 7].

Eyelid myokymia is a gentle muscle contraction of eyelid, generally affecting one eyelid (more often lower eyelid, but upper eyelids as well). The patients may feel that their eyelid is “jumping wildly,” but others do not notice the movement. Contractions are self-limited and episodic, lasting seconds to hours and sometimes to weeks [8]. The involvement of lower and upper eyelids on the same side or the involvement of eyelids on both sides of the face at the same time is rare. Myokymia is associated with fatigue, anxiety, stress, and exercise and excessive use of caffeine [9]. Medication induced myokymia is rare. Clozapine, gold salts, and flunarizine can induce myokymia [7]. In this case, I have presented a case of binge eating disorder suspected TPM-induced eyelid myokymia.

## 2. Case Presentation

A 47-year-old Iranian woman was referred to a psychiatric clinic complaining of distressing eyelid twitching called eyelid myokymia. She was taking TPM 50 mg HS for at least 16 months, to control her weight gain due to binge eating problem. She explained that the eyelid twitching started from lower right eyelid which was not so much disturbing at first but was aggravating with stress and fatigue and diminishing by rest, but in the previous month, there was no response to rest. She had searched through the Internet and found out that this was a benign, periodic, and self-limiting condition, but less response to resting made her get worried more and more in the last month.

Physical examination was normal except previously diagnosed mild hypertension, controlled with 25 mg/day losartan over a period of 2 years. MRI and laboratory tests revealed no abnormal findings. An ophthalmological consultation was carried out but reported no significant visual disturbance. The patient was reassured and asked to continue her medications and also advised to decrease work time and to rest more.

One month later, she came back with worsening of her eyelid twitching which was expanded to her upper eyelid and occasionally the same side eyebrow. Her self-esteem and social communication were severely affected because she believed that twitching can be observed by others. Physical examination was again normal and no new sign was detected. She was advised not to use TPM anymore, with possible diagnosis of TPM-induced myokymia.

Initially because of its favorable effects on weight loss, she resisted discontinuing TPM, but actually worsening of symptoms made her respect the physician's decision. The patient's symptoms decreased gradually and disappeared completely two weeks after TPM discontinuation. TPM discontinuation resulted in increasing appetite and body weight over two months; consequently she became depressed and started using TPM again. Eyelid myokymia reappeared two weeks later.

## 3. Discussion

The patient was not a smoker or excessive alcohol or caffeine abuser. Increasing the resting time and changing life style made no significant changes in her eyelid twitching. There was no definite evidence by neuroimaging and clinical or laboratory evaluations causing eyelid myokymia. Since losartan and TPM have no significant pharmacological interaction, myokymia cannot be explained on the basis of pharmacokinetic drug interactions. However, the possibility of pharmacodynamic interactions cannot be ruled out. Medrano-Martínez et al. [7] stated that it was not clear whether the eyelid myokymia was related to TPM or migraine in their study. Our patient had never complained of migraine.

Considering the temporal relationship between TPM administration and the appearance of myokymia, as well as alleviating and aggravating the symptoms with drug discontinuation and rechallenge, respectively, we considered eyelid myokymia a presumptive side effect of TPM.

The orbicularis oculi (OO) muscle is innervated unilaterally from the facial nucleus. The levator palpebrae superioris (LPS) muscle is innervated bilaterally from the central caudal part of the oculomotor nucleus. Primary sensory afferent nerves from the cornea and eyelid end in medullary spinal trigeminal nucleus. The pars caudalis of the spinal trigeminal nucleus sends excitatory projections to the OO motor neurons, ipsilaterally. The trigeminal nucleus sends excitatory projections to the OO motor neurons and inhibitory projections to the LPS, bilaterally. This is the proper circuitry for the trigeminal blink reflex. It occurs with simultaneous contraction of OO and inhibition of LPS [10, 11].

Any disorder that affects this close LPS-OO relationship leads to central lid movement disorders. Three groups of supranuclear motor impairment of lid movements are considered: the disorders of the lid-eye movements' coordination, the disorders of blinking and lid “postural” maintenance, and disorder of arbitrary lid movements [12–14]. Myokymia is localized and has a chronic process. The absence of other ipsilateral facial muscles involvement and chronic and localized nature of myokymia indicate that the disorder is peripheral [15].

TPM is a sulfa-derivative monosaccharide. Its mechanisms of action are blockage of voltage-gated sodium channels, hyperpolarization of potassium currents, suppression of the AMPA/kainite receptor, enhancement of postsynaptic GABA receptor activity, and mild inhibition of carbonic anhydrase isoenzymes [2, 7].

Autoimmune processes, genetic changes, radiation, demyelination, toxic effects, ischemia, and hypoxia affect motor neuron axons and produce myokymia. Voltage-gated potassium channels are sensitive to voltage changes in the cell's membrane potential. During action potentials, they return the depolarized cell to a resting condition. These channels have a key role in controlling neuronal excitability in peripheral, central, and autonomic nervous system [16, 17]. Hyperexcitability of peripheral nerves may have genetic, immunologic, toxic, and drug induced etiologies [7]. Although TPM acts on calcium and potassium channels related to myokymia, the definite mechanism of action is not well understood which calls for further researches.

## 4. Conclusion

Although eyelid myokymia is a benign and self-limited condition, it sometimes becomes a source of distress in chronic long standing cases. Possible side effects of drugs currently used by patients should be considered in clinical evaluation. Due to the expanding spectrum of indications for TPM, neurologists and psychiatrists should be aware of the ocular and neuroophthalmologic side effects of this drug.

## Abbreviations

TPM: Topiramate
HS: Hour of sleep
mg: Milligrams
LPS: Levator palpebrae superioris
OO: Orbicularis oculi
GABA: Gamma-amino butyric acid
AMPA: *α*-Amino-3-hydroxy-5-methyl-4-isoxazole propionic acid.

## References

[1] Privitera M. D., Brodie M. J., Mattson R. H., Chadwick D. W., Neto W., Wang S. (2003). Topiramate, carbamazepine and valproate monotherapy: double-blind comparison in newly diagnosed epilepsy. *Acta Neurologica Scandinavica*.

[2] Abtahi M. A., Abtahi S. H., Fazel F. (2012). Topiramate and the vision: a systematic review. *Clinical Ophthalmology*.

[3] Fleming J. W., McClendon K. S., Riche D. M. (2013). New obesity agents: lorcaserin and phentermine/topiramate. *Annals of Pharmacotherapy*.

[4] Singh J., Kumar R. (2015). Phentermine-topiramate: first combination drug for obesity. *International Journal of Applied and Basic Medical Research*.

[5] Ghaemi S. N., Manwani S. G., Katzow J. J., Ko J. Y., Goodwin F. K. (2001). Topiramate treatment of bipolar spectrum disorders: a retrospective chart review. *Annals of Clinical Psychiatry*.

[6] Stefan H., Hubbertz L., Peglau I. (2008). Epilepsy outcomes in elderly treated with topiramate. *Acta Neurologica Scandinavica*.

[7] Medrano-Martínez V., Pérez-Sempere A., Moltó-Jordá J. M. (2015). Eyelid myokymia in patients with migraine taking topiramate. *Acta Neurologica Scandinavica*.

[8] Oh S. J., Alapati A., Claussen G. C., Vernino S. (2003). Myokymia, neuromyotonia, dermatomyositis, and voltage-gated K^+^ channel antibodies. *Muscle and Nerve*.

[9] Rucker J. C. (2011). Chapter 15—normal and abnormal lid function. *Handbook of Clinical Neurology*.

[10] May P. J., Baker R. G., Chen B. (2002). The eyelid levator muscle: servant of two masters. *Movement Disorders*.

[11] May P. J., Porter J. D. (1998). The distribution of primary afferent terminals from the eyelids of macaque monkeys. *Experimental Brain Research*.

[12] Ross A. H., Elston J. S., Marion M.-H., Malhotra R. (2011). Review and update of involuntary facial movement disorders presenting in the ophthalmological setting. *Survey of Ophthalmology*.

[13] Esteban Á., Traba A., Prieto J. (2004). Eyelid movements in health and disease. The supranuclear impairment of the palpebral motility. *Neurophysiologie Clinique*.

[14] Helmchen C., Rambold H. (2007). The eyelid and its contribution to eye movements. *Developments in Ophthalmology*.

[15] Banik R., Miller N. R. (2004). Chronic myokymia limited to the eyelid is a benign condition. *Journal of Neuro-Ophthalmology*.

[16] Hart I. K., Maddison P., Newsom-Davis J., Vincent A., Mills K. R. (2002). Phenotypic variants of autoimmune peripheral nerve hyperexcitability. *Brain*.

[17] Shah N. H., Aizenman E. (2014). Voltage-gated potassium channels at the crossroads of neuronal function, ischemic tolerance, and neurodegeneration. *Translational Stroke Research*.

